# To Dr. Willan

**Published:** 1807-12

**Authors:** James Upton

**Affiliations:** King Street


### To Dr. WILLAN.

SIR,

IN your excellent, but unfinished work, on Cutaneous Diseases, I remark, that under the article Rubeola, you mention not having seen twice, in the same subject, the *febrile* Rubeola.

Although I admit the strong affinity subsisting between different RASHES, and the liability of medical men, sometimes, to mistake scarlatina and rubeola, especially when the former is attended with catarrhal symptoms; yet, I think, there are many exceptions to the general rule, having seen a mild and severe attack of rubeola in the same subject three or four times in the course of twenty years.

Last summer, the rubeola sine catarrho, appeared in a ladies boarding school at Clapham, and preceded by about a month the real disease. Mr. Hey, surgeon of Leeds, whose high professional character, if not personally, must be known to you. He has assured me, both by letter and in conversation, that this fact has occurred in his experience.

The enclosed case will, in some degree, illustrate what I have been endeavouring to explain, and which you are at liberty to use in any way you think proper.

I am, &c.

JAMES UPTON.

*King Street, October* 30, 1807.

### CASE.

Harriet Anne Upton, aged nine years, came home from school last Easter holidays, in apparent good health. Two or three days previous to her return, and to the expiration of her fortnight's recess, she looked heavy about the eyes, had a short frequent cough, with all that *crossness* attendant upon children's indisposition; sneezing and tenderness of the eyes succeeded, and on the third day, the 14th of her being at home, a considerable rash appeared, with great fever, and sensibility to light.

Upon enquiry respecting her school-fellows, she told me that on the morning of her leaving school, a young lady sat next to her at breakfast, who had a rash upon her face and arms.—The eruption on my child was barely perceptible on the third day from its first appearance, and all the other symptoms subsided. Being fully convinced her complaint complaint was the rubeola, I took an early opportunity of calling at the school, and on the attending surgeon Mr. Gardner, from whom I learned, that all the young ladies there, had had more or less of a rash, but without much catarrhal affection, and that none of them required either medicine or confinement. He was, therefore, doubtful respecting the nature of the disease, though he at first considered it to be the measles.

My little girl, in less than a month after her return to school, was again affected with the rubeola, in nearly the same manner as she had been on the former occasion.

